# Sipuleucel-T and Androgen Receptor-Directed Therapy for Castration-Resistant Prostate Cancer: A Meta-Analysis

**DOI:** 10.1155/2016/4543861

**Published:** 2016-12-12

**Authors:** Renliang Yi, Baoxin Chen, Peng Duan, Chanjiao Zheng, Huanyu Shen, Qun Liu, Chen Yuan, Weilin Ou, Zhiheng Zhou

**Affiliations:** ^1^Guangzhou Hospital of Guangzhou Military Region, Guangzhou, China; ^2^School of Public Health, Guangzhou Medical University, Guangzhou, China; ^3^Department of Surgery, Massachusetts General Hospital, Harvard Medical School, Boston, MA, USA

## Abstract

New treatments, such as sipuleucel-T and androgen receptor- (AR-) directed therapies (enzalutamide (Enz) and abiraterone acetate (AA)), have emerged and been approved for the management of castration-resistant prostate cancer (CRPC). There are still debates over their efficacy and clinical benefits. This meta-analysis aimed to investigate the efficacy and safety of sipuleucel-T and AR-directed therapies in patients with CRPC. RevMan 5.1 was used for pooled analysis and analysis of publication bias. Seven studies were included in the meta-analysis, with three studies in sipuleucel-T (totally 737 patients, 488 patients in treatment group, and 249 patients in placebo group) and four in AR-directed therapies (totally 5,199 patients, 3,015 patients in treatment group, and 2,184 patients in placebo group). Treatment with sipuleucel-T significantly improved overall survival in patients with CRPC and was not associated with increased risk of adverse event of grade ≥3 (*p* > 0.05). However, treatment with sipuleucel-T did not improve time-to-progression and reduction of prostate-specific antigen (PSA) level ≥50% was not significantly different from that with placebo. AR-directed therapies significantly improved overall survival in patients with CRPC and improved time-to-progression and reduction of PSA level ≥50%. AR-directed therapies did not increase risk of adverse event of grade ≥3 (*p* > 0.05).

## 1. Introduction

Prostate cancer is one of the most frequently diagnosed cancers in men. Worldwide, in 2015, it is the second most common newly diagnosed cancer and the fourth most common cause of cancer death in men. In the United States, incidence and mortality of prostate cancer ranked first and second, respectively, in men. Over the past few years, incidence of prostate cancer increased steadily, with slowly increased mortality [1–3]. Current treatments for prostate cancer include surgical and medically induced castration and androgen deprivation therapy (ADT) using androgen receptor (AR) antagonists [4]. Despite these treatments, a sizable number of patients will eventually experience disease recurrence and progression [5]. Castrate-resistant prostate cancer (CRPC) is defined as disease progression despite ADT and may present a spectrum of disease ranging from rising prostate-specific antigen (PSA) levels, progression of preexisting disease, or appearance of new metastases [6–8]. CRPC poses a great challenge in the management of prostate cancer.

Docetaxel, approved by the US Food and Drug Administration (FDA) in 2004, is a taxane drug that induces polymerization of microtubules and phosphorylation of Bcl-2 protein. Three weeks of combined docetaxel and prednisone is currently considered the standard of first-line chemotherapy for men with CRPC [9]. The second-line chemotherapy with cabazitaxel has been shown to increased survival time in patients with CRPC. However, severe adverse events have been reported for these treatments [10, 11]. With advances in the understanding of disease pathophysiology, new treatments for CRPC emerge in the recent years that aim to improve both survival and quality-of-life of patients [12]. These treatments include cancer immunotherapy such as sipuleucel-T, AR-directed therapies such as abiraterone acetate (AA) and enzalutamide (Enz), radium-223, and PROSTVAC [13–18]. Radium-223 is mainly used to manage bone metastases in CRPC [19]. Trials of immunotherapy of PROSTVAC which utilizes recombinant poxviruses to express PSA are ongoing [20]. Phase III clinical trials have been conducted for sipuleucel-T, AA, and Enz and FDA has approved their use in patients with CRPC [21–23]. These new treatments hold great potential as the first-line treatments for patients with CRPC. Finding the optimal regimen is now the major clinical challenge. This meta-analysis aimed to investigate and compare the efficacy and safety of these two treatments and to provide scientific evidence for the management of CRPC.

AA is a steroidal antiandrogen that exerts its effect through inhibiting CYP17A and it also acts as an antagonist of AR [24, 25]. Clinical trials showed significantly improved survival for treatment with AA compared with placebo. It was approved by the FDA in 2011 for patients with CRPC. Enz is a synthetic nonsteroidal pure antiandrogen. It has a strong binding affinity for AR and in addition prevents binding of AR to deoxyribonucleic acid and AR to coactivator proteins [26]. It was approved by the FDA in 2012 for patients with CRPC.

Sipuleucel-T (PROVENGE®) is an autologous vaccine. The antigen presenting cells (APCs) are harvested from individual patient's peripheral blood and later incubated with recombinant fusion protein antigen, which contains both prostatic acid phosphatase and granulocyte-macrophage colony-stimulating factor [27, 28]. This process activates the APCs, which are critical for priming a cytotoxic T-lymphocyte-mediated immune response [27]. These activated APCs are then reinfused into the individual patient. In 2010, sipuleucel-T became the first immune-therapeutic agent approved by the FDA for patients with CRPC, based on consistent observed improvement in overall survival.

This meta-analysis aimed to further determine the clinical efficacy and safety of these two types of treatments, namely, sipuleucel-T and AR-directed therapies (AA and Enz), in the management of CRPC. Survival and disease progression were assessed by overall survival (OS) and time-to-progression (TTP) [29], respectively. Biological endpoint was assessed as a ≥50% reduction of PSA level. Adverse events of grade ≥3 were also reviewed.

## 2. Methods

### 2.1. Literature Search and Study Selection

We systematically searched seven literature databases (OVID, Springer, PubMed, Web of Science, ScienceDirect, Medline, and Cochrane Library) from 1966 to October 2015 for all relevant articles by entering terms including “castrate-resistant prostate cancer”, “sipuleucel-T”, “enzalutamide”, and “abiraterone acetate” as key words, title, subject heading, and text word. We also searched for potentially missed articles from the reference list of retrieved articles and from previous narrative reviews on this topic.

Studies were included if they met the following criteria: (1) randomized double-blind place-controlled clinical trials of sipuleucel-T, AA, and Enz presenting original data; (2) patients with CRPC; (3) English articles published before October 2015. In case of duplicated reports, the article presenting the latest and the most comprehensive data on the largest cohorts was selected. Studies were excluded if (1) they were duplicated reports, were of poor quality, were lacking original data, or presented incomplete data; (2) they were review articles, conference abstracts, or commentary. Two authors (Renliang Yi and Baoxin Chen) conducted literature search and study selection independently. Results were compared and discrepancies were resolved by a discussion with another author (Peng Duan).

### 2.2. Quality Assessment

Full text of articles that fulfilled inclusion and exclusion criteria were retrieved for review. Quality of the included articles was assessed using the Newcastle-Ottawa Scale (NOS) [30], in which a study is judged on three broad perspectives: the selection of the study groups (adequate definition of the cases, representativeness of the cases, selection of controls, and definition of controls), the comparability of the groups (compatibility of cases and controls), and the ascertainment of the outcome of interest (ascertainment of exposure, ascertainment of cases and controls, and nonresponse rate). Total score of NOS is nine, with higher score indicating higher quality. Two authors (Qun Liu and Chen Yuan) conducted quality assessment independently. Results were compared and discrepancies were resolved by a discussion with another author (Weilin Ou).

### 2.3. Data Extraction

The following outcomes were extracted from each study: OS, TTP, reduction of PSA level ≥50%, and adverse events of grade ≥3. Two authors (Chanjiao Zheng and Huanyu Shen) conducted data extraction independently. Results were compared and discrepancies were resolved by a discussion with corresponding author (Zhiheng Zhou).

### 2.4. Data Synthesis and Analysis

Review Manager 5.1 software was used for data synthesis and analysis. The hazard ratio (HR) with 95% confidence interval (CI) was calculated for dichotomized data. Quantitative data were expressed as weighted mean difference (WMD) with 95% CI. Heterogeneity analysis was performed using *q* test with *p* > 0.1 and *I* ^2^ < 50% suggesting homogeneity among studies. For data without significant heterogeneity, fixed-effect models were used for pooled analyses. In case of significant heterogeneity, sensitivity analysis was performed by excluding the study with the highest variance. In the case that no definite cause was found for heterogeneity, random-effect model was used for pooled analyses. The significance of pooled data was further tested and a *p* < 0.05 was considered statistically significant. When enough studies were included, funnel plot was delineated and the publication bias was evaluated.

## 3. Results

### 3.1. Study Selection

**Figure 1:**
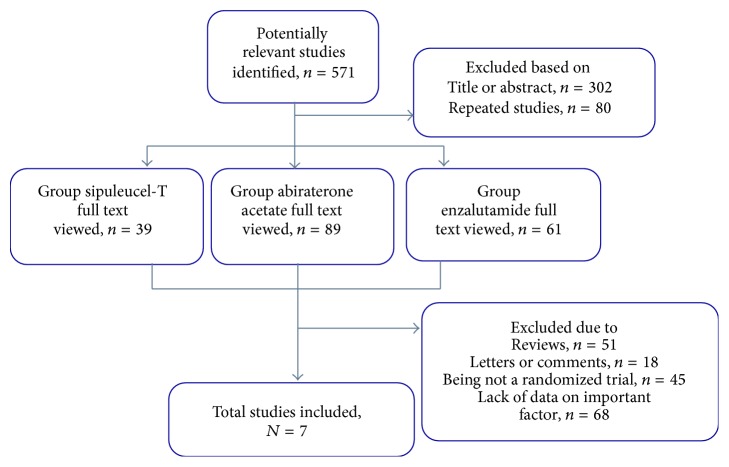
Flow chart of study selection. The summary of the study selection process was shown by flow chart.

Our search resulted in 571 articles. A total of 302 articles were excluded after reviewing titles and abstracts, and 80 articles were excluded due to duplicated reports. A total of 182 articles were further excluded after full-text review. Exclusion reasons included review articles and commentary, correspondence, nonrandomized placebo-controlled trials, and lack of complete study outcomes. Seven articles were included for the meta-analysis: three articles on sipuleucel-T, two on AA, and two on Enz, respectively.  Figure 1 shows the flow of literature search and study selection.

### 3.2. Study Characteristics

All seven studies were randomized, double-blind, placebo-controlled clinical trials. Results of quality assessment using NOS for the seven studies are showed in Table 1. The seven studies included a total of 5,936 patients with CRPC.  Table 2 shows the main characteristics of the included studies. Regimens used in these studies were as follows: (1) sipuleucel-T: patients were randomly assigned in a 2 : 1 ratio to receive either sipuleucel-T or placebo every two weeks, for a total of three infusions; (2) AA: intervention group received combined AA 1000 mg and prednisone 10 mg daily and placebo group received prednisone 10 mg daily plus placebo; (3) Enz: intervention group received Enz 60 mg daily and placebo group received placebo.

### 3.3. Overall Survival (OS)

**Figure 2:**
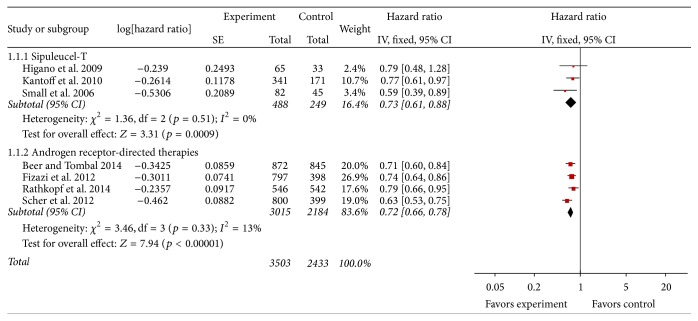
Forest plot of hazard ratio of overall survival of sipuleucel-T and androgen receptor-directed therapies compared with placebo in men with castration-resistant prostate cancer. The bars with squares in the middle represent 95% confidence intervals (95% CIs) and HRs. The central vertical solid line indicates the HRs for null hypothesis. The size of the diamonds represents the weight for the random-effect model in the meta-analysis.

All seven studies provided data on survival with follow-up period up to 36 months [29, 31–36]. Analyses of OS were performed in 5,936 patients, with 737 patients for sipuleucel-T (intervention group versus placebo group: 488 versus 249 patients) and 5,199 patients for AR-directed therapies (intervention group versus placebo group: 3,015 versus 2,184 patients).  Figure 2 shows the forest plot of analysis of OS. Results showed that, compared with placebo, both sipuleucel-T and AR-directed therapies significantly improved survival of patient with CRPC. Pooled HR for OS was 0.73 for sipuleucel-T (95% CI: 0.61–0.88; *Z* = 3.31; *p* < 0.001) and 0.72 for AR-directed therapies (95% CI: 0.66–0.78; *Z* = 7.94; *p* < 0.00001). Tests for heterogeneity showed insignificant results, indicating homogeneity among studies (both *p* > 0.1 and both *I* ^2^ < 50%).

### 3.4. Time-to-Progression (TTP)

**Figure 3:**
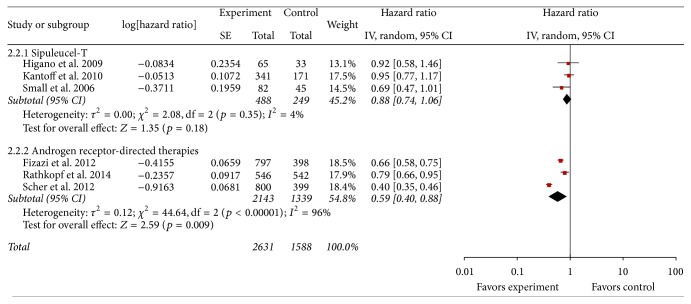
Forest plot of hazard ratio of time-to-progression of sipuleucel-T and androgen receptor-directed therapies compared with placebo in men with castration-resistant prostate cancer. The bars with squares in the middle represent 95% confidence intervals (95% CIs) and HRs. The central vertical solid line indicates the HRs for null hypothesis. The size of the diamonds represents the weight for the random-effect model in the meta-analysis.

Six studies with a total of 5,936 patients reported TTP [29, 31–35], including 737 patients for sipuleucel-T (intervention group versus placebo group: 488 versus 249 patients) and 5,199 patients for AR-directed therapies (intervention group versus placebo group: 3,015 versus 2,184 patients).  Figure 3 shows the forest plot of analysis of TTP. Compared with placebo, sipuleucel-T showed no significant favorable effect on TTP with pooled HR of 0.88 (95% CI: 0.74–1.06; *Z* = 1.35; *p* = 0.18). Test for heterogeneity was not significant (*p* = 0.35, *I* ^2^ = 4%). In contrast, AR-directed therapies showed significant improvement in TTP with pooled HR of 0.59 (95% CI: 0.40–0.88; *Z* = 2.59; *p* = 0.009).

### 3.5. Reduction of PSA Level ≥50%

**Figure 4:**
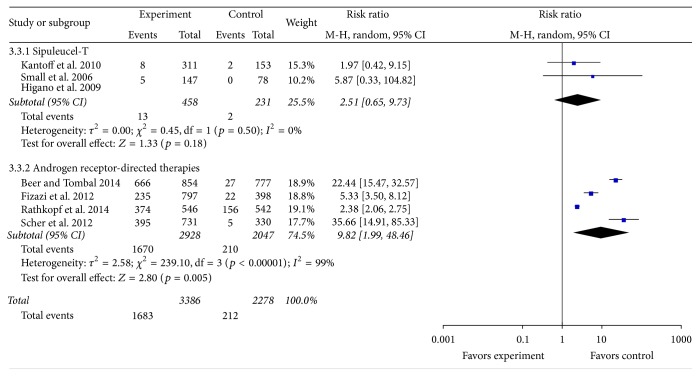
Forest plot of risk ratio of reduction of prostate-specific antigen ≥50% of sipuleucel-T and androgen receptor-directed therapies compared with placebo in men with castration-resistant prostate cancer. The bars with squares in the middle represent 95% confidence intervals (95% CIs) and RRs. The central vertical solid line indicates the RRs for null hypothesis. The size of the diamonds represents the weight for the random-effect model in the meta-analysis.

Seven studies with a total of 5,936 patients reported reduction of PSA level ≥50% as study outcome [29, 31–36], including 689 patients for sipuleucel-T (intervention group versus placebo group: 458 versus 231 patients) and 4,975 patients for AR-directed therapies (intervention group versus placebo group: 2,928 versus 2,047 patients). Pooled RR showed that sipuleucel-T has no significant effect on reducing PSA level ≥50% (RR: 2.51; 95% CI: 0.65–9.73; *Z* = 1.33; *p* = 0.18). Test for heterogeneity was not significant (*p* = 0.5; *I* ^2^ = 0%). In contrast, AR-direct therapies showed significant effect on reducing PSA level ≥50% (RR: 9.82; 95% CI: 1.99–48.46; *Z* = 2.89; *p* = 0.004) (Figure 4).

### 3.6. Adverse Events (Grade ≥3)

**Figure 5:**
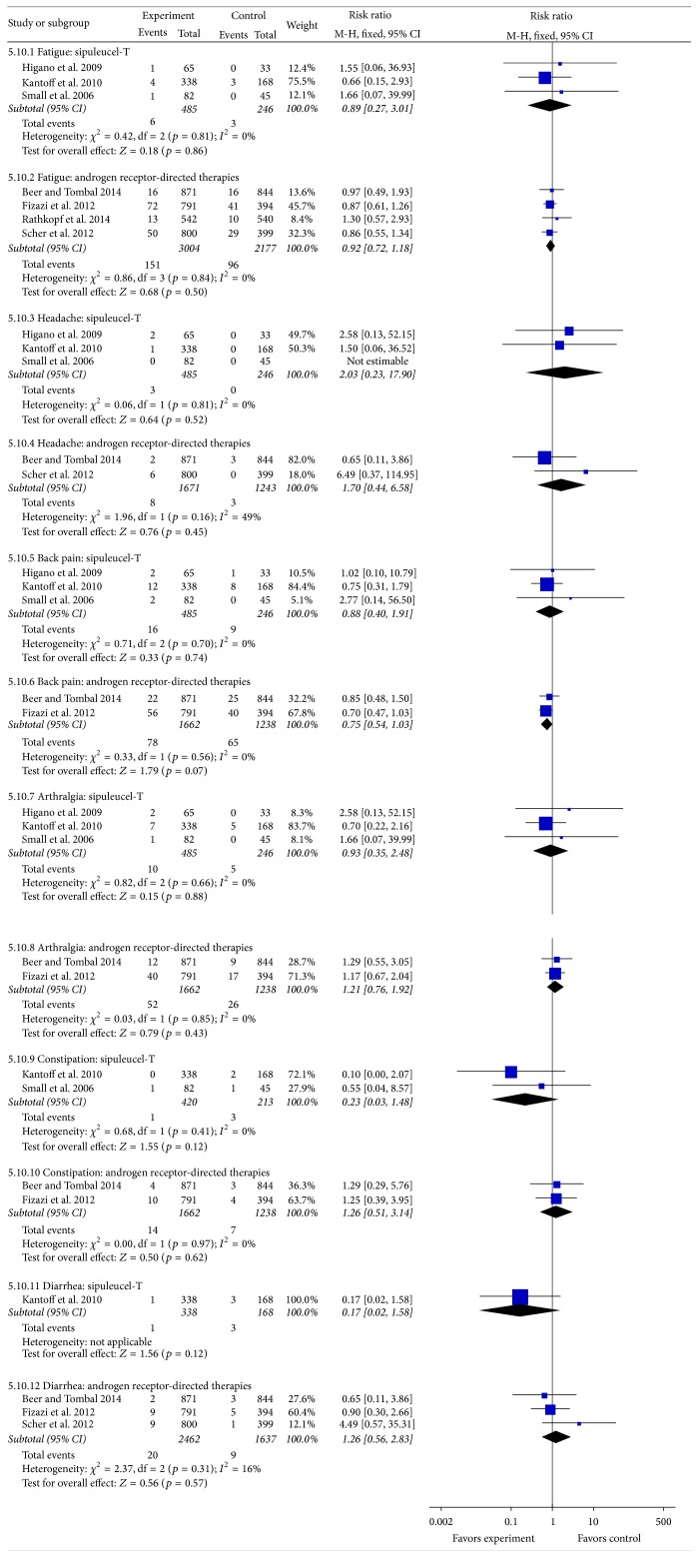
Forest plot of risk ratio of all adverse events of grade ≥3 of sipuleucel-T and androgen receptor-directed therapies compared with placebo in men with castration-resistant prostate cancer. The bars with squares in the middle represent 95% confidence intervals (95% CIs) and RRs. The central vertical solid line indicates the RRs for null hypothesis. The size of the diamonds represents the weight for the random-effect model in the meta-analysis.

To investigate the safety of these treatments, we compared the occurrence of adverse events of grade ≥3, including fatigue, headache, back pain, arthralgia, constipation, and diarrhea, with that in placebo. Pooled RR revealed that, compared with placebo, risk of adverse event was not significantly increased for sipuleucel-T and AR-directed therapies (*p* > 0.05, Table 3 and Figure 5). There were also no significant adverse events related to these two treatments.

### 3.7. Publication Bias

**Figure 6:**
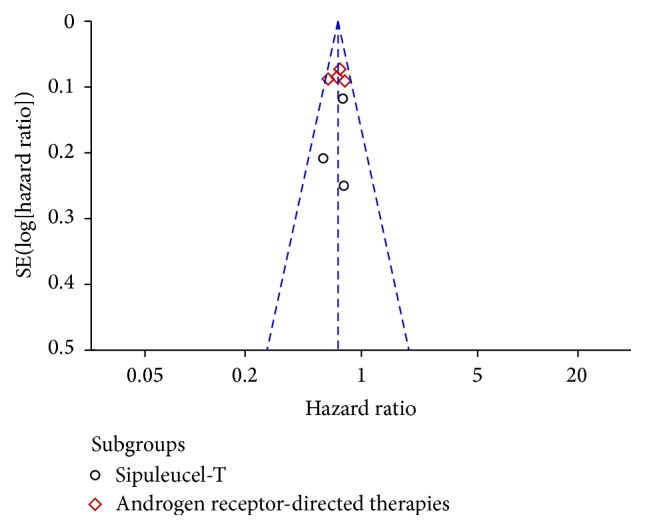
Funnel plot on overall survival for all included studies. The funnel graph plots the log of HR against the standard error of the log of the OR. The circles indicate the individual studies in the meta-analysis. The line in the center represents the meta HR.

Funnel plot for publication bias was performed on study outcome of OS.  Figure 6 shows symmetry funnel plot, indicating that there was no significant evidence of publication bias.

## 4. Discussion

Researches on novel treatments for CRPC have gained increasing interest in the past few years, especially those on sipuleucel-T and AR-directed therapies. This meta-analysis investigated the efficacy and safety of sipuleucel-T and AR-directed therapies, providing valuable information that might be useful clinical evidence on the treatments for CRPC. We found that both sipuleucel-T and AR-directed therapies could significantly improve OS in patients with CRPC, with favorable safety. AR-directed therapies appear to have superior effects in improving TTP and in reduction of PSA level. However, there are still debates over the efficacy and optimal regimen of these new treatment methods.

It has been known that traditional chemotherapeutic drugs lacked the selectivity on target tumor cells, which may cause different damage to normal cells, or even serious effects on patients. For example, Lim et al. indicated that adverse effects of docetaxel including edema, neurotoxicity, and hair loss limit its application [37]. Zhou et al. also showed 2.7% of CRPC patients died after docetaxel plus prednisone therapy, 58.56% had neutropenia, and 19.82% had leukopenia [38]. Unlike the traditional ones, sipuleucel-T and AR-directed therapies target tumor cells, thus causing little toxic effects on normal cells due to high selectivity. For instance, AA and Enz antagonize androgen receptors to inhibit the activity of tumor cells. Similarly, sipuleucel-T could elicit immune response targeting against antigen prostatic acid phosphatase (PAP) that is highly expressed in most prostate cancer cells [39, 40].

Many results showed that sipuleucel-T and AR-directed therapies improved the overall survival [29, 31–36] by exerting different effects on TTP, PSA level, and AEs. Although surgery, radiotherapy, or chemotherapy had stopped for a period of time before the new drugs clinical trials were given, it is still hard to rule out the possibility of the influence by the former treatments. Therefore, further clinical validation is needed. Beyond that, we paid more attention to the sequela, applicable scopes, and contraindications of these treatments.

All trials had strict selection of patients. To be an eligible case for sipuleucel-T trial, histological confirmation on castrate-resistant prostate cancer, serum testosterone level <50 ng/dL, and a considerable somatic function for expected survival were required. Patients accepted for AA trial should had no more than two previous chemotherapies, at least one previous docetaxel therapy, and mild symptoms or no symptoms (radiographic progression in soft tissue or bone with or without PSA progression, PSA <50 ng/dL, and ECOG performance status of 2 or less). And Enz had similar requirement to AA's.

Upon the drug usage, patients from sipuleucel-T trials were scheduled to undergo leukapheresis procedures every 2 week for a total of three times, and on the second day after each leukapheresis procedure, patients were treated by infusion of sipuleucel-T or placebo. Patients from AA trials received abiraterone acetate 1000 mg once daily plus prednisone 5 mg twice daily by oral or placebo plus prednisone. And in Enz trials patients received enzalutamide 160 mg orally once daily or matched placebo.

We found that in AA trials patients with lower score of ECOG, age ≥ 65 years, and PSA level > or < median had the higher HR. On the contrary, in enzalutamide trials, patients with age ≥ 65 years, higher score of ECOG, and PSA Level > median had the higher HR. While in sipuleucel-T trials, patients with age < median, psa < or > median, and higher score of ECOG had the higher HR. Such differences may be related to the characteristics of different individuals. Drake 2012 indicated that the subgroup of patients aged less than 65 years did not favor sipuleucel-T. Another observation by them was the potential harm from the IMPACT study interventions, because, to some extent, sipuleucel-T broke the immune balance [41].

Challenges remain in finding the optimal regimen for sipuleucel-T and AR-directed therapies. Combined sipuleucel-T, AA, and prednisone formula has been proposed as a novel treatment diagram in CRPC. Research has shown that concurrent administration of AA and prednisone did not blunt immunologic effects or alter immune parameters that correlate with sipuleucel-T's clinical benefits [42]. Cumulative APC activation, cumulative APC number, total nucleated cell counts, and immune responses to sipuleucel-T were not affected by coadministration of AA and prednisone. Such combination of treatments was well tolerated, with no new risk marker emerging. Sipuleucel-T is recommended as the first-line treatment for patients with CRPC by the National Comprehensive Cancer Network [43] and it is recommended as early use in asymptomatic CRPC or patients with mild symptoms. Comparatively, treatments with AA and Enz could induce symptomatic disease progression. Badrising et al. 2014 reported that tolerance could be built up when combining Enz and AA [44]. This was possibly a result of mutation of AR induced by prednisone, which will subsequently impact the effect of Enz on AR. Therefore, it appears that there is limited clinical benefit for combination or sequential use of Enz and AA [45–48].

## Figures and Tables

**Table 1:** Quality indicators by Newcastle-Ottawa Scale.

Studies	Selection				Comparability		Exposure			Score
	(1)	(2)	(3)	(4)	(5A)	(5B)	(6)	(7)	(8)	
Beer and Tombal, 2014	Yes	Yes	Yes	Yes	No	Yes	Yes	Yes	Yes	8
Fizazi et al., 2012	Yes	Yes	Yes	Yes	Yes	Yes	Yes	Yes	Yes	9
Higano et al., 2009	Yes	Yes	Yes	Yes	No	Yes	Yes	Yes	Yes	8
Kantoff et al., 2010	Yes	Yes	Yes	Yes	Yes	Yes	Yes	Yes	Yes	9
Rathkopf et al., 2014	Yes	Yes	Yes	Yes	Yes	Yes	Yes	Yes	Yes	9
Scher et al., 2012	Yes	Yes	Yes	Yes	No	Yes	Yes	Yes	Yes	8
Small et al., 2006	Yes	Yes	Yes	Yes	No	Yes	Yes	Yes	Yes	8

(1): case independent validation; (2): representativeness of the cases; (3): community or hospital controls; (4): history of disease; (5A): study controls for the most important factor; (5B): study controls for any additional factor; (6): ascertainment of exposure; (7): was follow-up long enough for outcomes to occur? (8): adequacy of follow-up of cohorts.

**Table 2:** Main characteristics of included studies.

Study	*N*	Patients	Design	Interventions	Primary endpoint
Small et al., 2006	127	CRPC	Randomized, double-blind, placebo-controlled	Sipuleucel-T, placebo	OS, TTP, reduction of PSA > 50%, AEs grade ≥ 3
Higano et al., 2009	98	CRPC	Randomized, double-blind, placebo-controlled	Sipuleucel-T, placebo	
Kantoff et al., 2010	512	CRPC	Randomized, double-blind, placebo-controlled	Sipuleucel-T, placebo	
Fizazi et al., 2012	1195	CRPC	Randomized, double-blind, placebo-controlled	Abiraterone acetate, placebo	
Rathkopf et al., 2014	1088	CRPC	Randomized, double-blind, placebo-controlled	Abiraterone acetate, placebo	
Scher et al., 2012	1199	CRPC	Randomized, double-blind, placebo-controlled	Enzalutamide, placebo	
Beer and Tombal, 2014	1717	CRPC	Randomized, double-blind, placebo-controlled	Enzalutamide, placebo	

CRPC: castration-resistant prostate cancer; OS: overall survival; TTP: time-to-progression; PSA: prostate-specific antigen; AEs: adverse events.

**Table 3:** Analyses of adverse events (grade ≥ 3).

Adverse events	References	Relative risk (95% confidence interval)	*p*	Heterogeneity
Sipuleucel-T				
Fatigue	Small et al., 2006 Higano et al., 2009 Kantoff et al., 2010	0.89 (0.27–3.01)	0.86	*p* = 0.81, *I* ^2^ = 0%
Headache		2.03 (0.23–17.9)	0.52	*p* = 0.81, *I* ^2^ = 0%
Back pain		0.88 (0.4–1.91)	0.74	*p* = 0.7, *I* ^2^ = 0%
Arthralgia		0.93 (0.35–2.48)	0.88	*p* = 0.66, *I* ^2^ = 0%
Constipation		0.23 (0.03–1.48)	0.12	*p* = 0.41, *I* ^2^ = 0%
Diarrhea		0.17 (0.02–1.58)	0.12	—
Androgen receptor-directed therapies				
Fatigue	Fizazi et al., 2012 Scher et al., 2012 Beer and Tombal, 2014 Rathkopf et al., 2014	0.92 (0.49–1.93)	0.5	*p* = 0.84, *I* ^2^ = 0%
Headache		1.7 (0.44–6.58)	0.45	*p* = 0.16, *I* ^2^ = 49%
Back pain		0.75 (0.54–1.03)	0.07	*p* = 0.56, *I* ^2^ = 0%
Arthralgia		1.21 (0.76–1.92)	0.43	*p* = 0.85, *I* ^2^ = 0%
Constipation		1.26 (0.51–3.14)	0.62	*p* = 0.97, *I* ^2^ = 0%
Diarrhea		1.26 (0.56–2.83)	0.57	*p* = 0.31, *I* ^2^ = 16%
